# Targeting Mitochondrial Fission Produces Both Neuroprotective and Detrimental Effects in the SOD1 Mouse Model of Amyotrophic Lateral Sclerosis

**DOI:** 10.3390/biology15161334

**Published:** 2026-08-07

**Authors:** Maria Ciuro, Chantal Rovetto, Angela A. Sirna, Salvatore Giunta, Giampiero Leanza, Rosario Gulino

**Affiliations:** 1 Department of Biomedical and Biotechnological Sciences, University of Catania, Via Santa Sofia 89, 95123 Catania, Italy; ciuromaria@hotmail.it (M.C.); chantal.rovetto23@gmail.com (C.R.); sirnangelalessia@gmail.com (A.A.S.); salvatore.giunta@unict.it (S.G.); 2 Department of Drug and Health Sciences, University of Catania, Via Santa Sofia 64, 95125 Catania, Italy; gpleanza@unict.it

**Keywords:** amyotrophic lateral sclerosis, dynamin-related protein 1, Mdivi-1, mitochondrial dynamics, motor neurons, physiology, skeletal muscle atrophy, SOD1^G93A^ mouse model, synaptic plasticity, TAR DNA-binding protein 43

## Abstract

Amyotrophic lateral sclerosis (ALS) is a neuromuscular disease characterized by progressive motor neuron (MN) degeneration and severe skeletal muscle atrophy. Despite extensive research, the mechanisms driving disease onset and progression remain incompletely understood. While MN loss is a defining feature of ALS, increasing evidence indicates that mitochondrial dysfunction contributes to disease pathogenesis. Here, we investigated the hypothesis that Mdivi-1, a pharmacological inhibitor of mitochondrial fission protein Drp-1, may exert neuroprotective properties in the SOD1^G93A^ mouse model of ALS. Treatment was initiated prior to symptomatic onset to assess its potential disease-modifying effects. Mdivi-1 administration resulted in partial preservation of spinal MNs, however, this benefit did not translate into functional improvement. Moreover, treated animals exhibited exacerbated muscle atrophy, increased cytoplasmic localization of TDP-43 in MNs and compromised synaptic plasticity. Drp-1 expression was reduced in SOD1 mice and further decreased following Mdivi-1 treatment, suggesting that mitochondrial dynamics may already be compromised in this model. Overall, our results also highlight possible off-target effects of Mdivi-1 and point to a context-dependent role of mitochondrial dynamics in ALS.

## Simple Summary

Amyotrophic lateral sclerosis (ALS) is a neurodegenerative disease that affects motor neurons, leading to progressive muscle weakness and severe muscle wasting. In addition to neuronal loss, increasing evidence suggests that problems in mitochondrial function, the process by which cells produce energy, may contribute to disease development and progression. In this study, we investigated the effects of Mdivi-1, a drug that inhibits mitochondrial fragmentation, in a mouse model of ALS. Treatment began before symptoms appeared in order to assess whether it could slow disease progression. Our findings showed that Mdivi-1 partially protected motor neurones in the spinal cord, but this did not result in improved motor performance. Moreover, treated animals developed more severe muscle wasting and changes in muscle fiber composition. We also observed cellular alterations associated with impaired neuromuscular function. Overall, these results highlight that mitochondrial dynamics play a complex and context-dependent role in the disease. A better understanding of these mechanisms will be important for developing more effective therapeutic strategies for ALS.

## 1. Introduction

Mitochondrial dysfunction is a prevalent feature of many neurodegenerative diseases including motor neuron (MN) disorders such as amyotrophic lateral sclerosis (ALS) [1,2]. Disruption of mitochondrial structure, dynamics, bioenergetics and calcium buffering has been extensively reported in ALS patients and model systems and has been suggested to be directly involved in disease pathogenesis [3,4]. ALS is a rapidly progressive and fatal disease in which the degeneration of upper and lower MNs leads to progressive muscle weakness, loss of motor function, and ultimately paralysis [5]. Despite extensive research, therapeutic options remain extremely limited, and no available treatment substantially modifies the disease progression [6]. Approximately 10% of ALS cases are familial, typically inherited in an autosomal dominant manner, while the remaining 90% are sporadic and do not show a clear genetic origin [5]. To date, more than 20 genes have been implicated in ALS, including superoxide dismutase 1 (SOD1), TAR DNA-binding protein 43 (encoding TDP-43), Fused in Sarcoma (FUS), and Chromosome 9 open reading frame 72 (C9orf72) [7]. Mutations in these genes collectively explain about 60% of familial cases and approximately 11% of sporadic cases [8]. The genes associated with ALS are involved in a broad spectrum of cellular functions, such as RNA processing, protein homeostasis, cytoskeletal dynamics, and oxidative stress responses [9]. This functional diversity highlights the complexity of the disease and suggests that multiple molecular mechanisms contribute to MN degeneration. Consequently, ALS is widely considered a multifactorial disorder in which genetic susceptibility interacts with additional cellular and possibly environmental factors to drive disease onset and progression [10].

Many of the identified ALS genes have a role in mitochondrial-related functions, highlighting the central role of mitochondrial biology in disease pathogenesis with accumulating evidence gathered from in vitro and in vivo disease models, suggesting primary dysfunction of mitochondria as a core ALS disease component [11,12,13]. Mitochondria are highly dynamic organelles that undergo continuous cycles of fission and fusion, processes that are tightly coordinated to preserve the mitochondrial network architecture. In mammalian cells, mitochondrial fission is primarily orchestrated by the cytosolic GTPase Dynamin-related protein 1 (Drp-1), which is recruited to the outer mitochondrial membrane, where it drives membrane scission. Beyond its canonical role in mitochondrial division, Drp-1 is essential for the spatial redistribution of mitochondria along axons and dendrites, ensuring adequate energy supply at synaptic sites [14,15,16,17].

Excessive mitochondrial fragmentation by Drp-1 has emerged as a critical pathogenic mechanism, increasingly implicated in a range of neurodegenerative disorders [18,19]. In this context, pharmacological and peptide-based approaches aimed at inhibiting mitochondrial fission have provided important experimental insights. Suppression of Drp-1 activity using small molecule inhibitor or disruption of the Drp-1-FIS1 interaction has been shown to reverse mitochondrial fragmentation [20,21], emerging as a promising neuroprotective compound. Although initially investigated for its anticancer properties, Mitochondrial division inhibitor 1 (Mdivi-1) has demonstrated protective effects in several neurodegenerative contexts, including Alzheimer’s disease models, as well as in experimental settings related to Parkinson’s disease, Huntington’s disease, and mouse models of brain injury [22,23,24,25].

However, whether these protective effects can be effectively translated to ALS remains unclear [26]. In particular, the overall impact of mitochondrial fission inhibition on disease onset and progression in ALS has not yet been fully elucidated. In this context, our laboratory previously demonstrated that Mdivi-1 attenuates functional deficits in a model of selective MN loss induced by intramuscular injection and retrograde transport of Cholera Toxin B-subunit conjugated to Saporin (CTB-Sap). While this model does not recapitulate the full complexity of ALS, it provides a controlled system to investigate MN degeneration and compensatory plasticity [27]. In this setting, Mdivi-1 treatment reduced denervation-associated impairments, enhanced synaptic plasticity, and promoted motor recovery, supporting the potential relevance of targeting mitochondrial dynamics in MN disorders.

Nevertheless, it has been suggested that the effects of Mdivi-1 appear to be context dependent, and its mechanisms of action are not entirely specific to Drp-1 inhibition, as additional effects on mitochondrial function and cellular homeostasis have been reported [28]. Recent evidence also indicates that its pharmacological profile extends beyond Drp-1 inhibition, involving additional actions on oxidative metabolism, including partial inhibition of Complex I [29]. This dual mechanism of action underscores both the therapeutic potential of Mdivi 1 and the need for careful interpretation of its effects in neurodegenerative disease models. These considerations raise important concerns regarding the importance of evaluating the effect of Mdivi-1 in a more complex and disease-relevant experimental model in the long-term.

Several in vitro studies, including models expressing the SOD1^G93A^ mutation, have shown that Mdivi-1 partially restores mitochondrial morphology and dynamics, supporting a role for excessive mitochondrial fission in ALS-related cellular dysfunction [30,31,32]. However, these findings remain incomplete, as many studies rely on simplified cellular systems or focus on specific compartments, such as skeletal muscle, rather than addressing the full complexity of MN degeneration and neuromuscular interactions in vivo. These limitations suggest that, while modulation of mitochondrial dynamics and bioenergetics can confer measurable benefits at the cellular level, such interventions are unlikely to fully counteract the multifactorial nature of ALS pathology. Rather, their overall impact may be partial and strongly context-dependent, underscoring the need for validation in more integrated and disease-relevant experimental models. Building on these findings, the present study investigated the impact of Mdivi-1 in the SOD1^G93A^ mouse model, one of the most widely used and well-characterized transgenic models of ALS. This model expresses a mutant form of human SOD1 and recapitulates several key features of the human disease, including progressive motor impairment, spinal MN degeneration, gliosis, and neuromuscular junction disruption [33,34].

## 2. Materials and Methods

### 2.1. Animals, Experimental Groups and Treatments

All experiments were conducted in accordance with the European Communities Council Directive 2010/63/EU, the ARRIVE guidelines and Italian regulations (D.Lgs. 26/2014). The study was approved by the local animal welfare committee (OPBA, University of Catania, Catania, Italy) and authorized by the Italian Ministry of Health (Authorization no. 793/2023-PR). Every effort was made to ensure compliance with the 3Rs principle. SOD1^G93A^ transgenic mice (B6.Cg-Tg(SOD1*G93A)1Gur/J) were obtained from The Jackson Laboratory (Bar Harbor, ME, USA) and housed at the Center for Advanced Preclinical in vivo Research (CAPiR, University of Catania) Transgenic hemizygous males were crossed with C57BL/6 females (Charles River Laboratories Italia s.r.l., Calco, Italy), both maintained on a C57BL/6 background. At approximately 5–6 weeks, animals were weaned and housed in groups (*n* ≤ 5 per cage, in IVC cages) under controlled temperature (23–25 °C), a 12/12 h light–dark cycle, with ad libitum access to food and water, and environmental enrichment.

After genotyping, age-matched female mice were randomly allocated to experimental groups (by a random number generator) as follows: SOD1 plus Mdivi-1 (SOD1-Mdivi1; *n* = 17), SOD1 plus vehicle (SOD1-Veh; *n* = 13), and wild type littermates receiving vehicle (WT-Veh; *n* = 17) or Mdivi-1 (WT-Mdivi1; *n* = 5). Treatments were administered intraperitoneally once per week starting at 12 weeks of age and continued for a total of 8 weeks. Body weight was recorded weekly to monitor general health status and treatment tolerance. Mdivi-1 (30 mg/kg) was dissolved in dimethyl sulfoxide vehicle (DMSO) at a concentration of 10.0 mg/mL, so as to deliver 3 μL of solution per gram of body weight.

At 20 post-natal weeks, all mice were euthanized. For histological analyses, animals were perfused with saline followed by ice-cold 10% formalin solution. Gastrocnemius muscles and spinal cords were dissected out and post-fixed in formalin for 24 h at 4 °C; spinal cords were subsequently cryoprotected in buffered 20% sucrose, embedded in Optical Cutting Temperature (OCT) compound (Bio-Optica, Milano, Italy), and sectioned at 30 µm using a freezing microtome (Microm KS-34, ThermoFisher Scientific, Waltham, MA, USA), whereas skeletal muscles were processed for paraffin embedding and sectioning.

For Western blotting analysis, spinal cords were rapidly dissected without perfusion, snap-frozen in dry ice, and stored at −80 °C pending protein extraction.

### 2.2. Genotyping

Genomic DNA was isolated from mouse tail biopsies. Tail samples were incubated overnight at 55 °C in STE lysis buffer (100 mM Tris-HCl pH 8.5, 5 mM EDTA, 0.2% SDS, 200 mM NaCl) containing Proteinase K. Following centrifugation, DNA was precipitated from the collected supernatant using isopropanol, air-dried, and subsequently resuspended in TE buffer (10 mM Tris-HCl, 1 mM EDTA, 10 mM NaCl). To facilitate complete resuspension, samples were further incubated at 55 °C. Genotyping for the SOD1^G93A^ mutant transgene was performed by PCR using PCR Master Mix (ThermoFisher Scientific, Waltham, MA, USA) and the following primer sequences: SOD1 forward 5′-CATCAGCCCTAATCCATCTGA-3′ and SOD1 reverse 5′-CGCGACTAACAATCAAAGTGA-3′.

### 2.3. Motor Tests

Global motor coordination, balance, and gait function were evaluated using the rotarod and grid walk assays. Behavioral assessments started at 13 weeks of age and were performed once per week over a 6-week period. Before the experimental protocol began, mice completed two habituation sessions in order to become accustomed to the apparatus and reduce stress-induced variability.

For the rotarod assay, animals were placed on an accelerating rotating rod programmed to increase from 18 to 30 rpm within 360 s, and the latency to fall was measured as an indicator of motor ability. Gait performance and limb placement accuracy were assessed with the grid walk test. During each trial, mice crossed a 40 cm runway consisting of round metal bars (4 mm in diameter) positioned 1.5 cm from each other. Sessions were video-recorded using a camera positioned parallel to the grid, and videos were subsequently analyzed offline (blinded) with KMPlayer software (version 2023.6.29.12). The number of footfalls in each trial were counted and averaged across animals.

### 2.4. Histology and Immunofluorescence

Longitudinal spinal cord sections were subjected to free-floating immunofluorescence staining. To remove residual OCT compound, sections were first rinsed in PBS and then blocked for 1 h at room temperature (RT) in PBS containing 5% normal donkey serum (NDS) and 0.4% Triton X-100. Subsequently, sections were incubated overnight with primary antibodies diluted in PBS supplemented with 2% NDS and 0.3% Triton X-100. The following primary antibodies were employed: goat polyclonal anti-choline acetyltransferase (anti-ChAT; Millipore, Merck Group, Darmstadt, Germany; Cat# AB144P, RRID: AB_2079751; 1:100) and rabbit monoclonal anti-TAR DNA-binding protein 43 (anti-TDP43 D9R3L; Cell Signaling Technology, Danvers, MA, USA; Cat# 89789S, RRID: AB_2631310; 1:100). Following PBS washes, sections were incubated for 60 min at RT with species-appropriate secondary antibodies diluted 1:500 in PBS containing 1% NDS and 0.3% Triton X-100. The secondary antibodies used were donkey anti-goat Alexa Fluor 546 (Invitrogen, ThermoFisher Scientific, Waltham, MA, USA; Cat# A-11056, RRID: AB_142628) and donkey anti-rabbit Alexa Fluor 488 (Invitrogen, ThermoFisher Scientific, Waltham, MA, USA; Cat# A21206, RRID: AB_2535792). Cell nuclei were counterstained with DAPI (1:1000; Invitrogen) for 10 min. Sections were mounted on glass slides and coverslipped using BrightMount mounting medium (Abcam, Cambridge, UK; Cat# ab103746). Fluorescence images were acquired with a Nikon fluorescence microscope (Nikon Europe B.V., Amstelveen, The Netherlands) and analyzed using ImageJ software v.1.54d (NIH, Bethesda, MD, USA).

Immunofluorescence analyses were performed on at least 3–4 sections/animal obtained from 5 to 6 animals per group. Quantification of ChAT-positive motor neurones (MNs) in the spinal cord ventral horn was carried out using QuPath software (v.0.7.0; https://qupath.github.io), by two independent investigators blind to the treatment. MN number was estimated by the average number of ChAT-positive MN profiles in at least 20–30 identical regions of interest (ROIs) per section, and normalizing values to WT controls. Subcellular localization of TDP-43 was quantitatively assessed in ImageJ by calculating the Manders’ colocalization coefficient (M1), representing the proportion of TDP-43 fluorescence signal overlapping with ChAT-positive cytoplasmic compartments, in at least 20–30 ROIs/section.

Gastrocnemius muscles were collected, fixed in formalin, and weighed. Muscle mass was normalized to body weight to provide an estimate of muscle atrophy while limiting the impact of global body weight loss in symptomatic mice. For muscle fiber morphology analysis, formalin-fixed gastrocnemius muscles were dehydrated and embedded in paraffin (Bio Optica Milano S.p.A., Milano, Italy) by incubation at 60 °C for 1.5 h. Paraffin blocks were sectioned at 5 µm thickness and stained with haematoxylin and eosin (H&E) for histopathological evaluation. Analyses were conducted on 3–4 sections per animal from 5 to 6 animals per group. Slides were digitized using a ScanScope digital scanner (Aperio, Bristol, UK), and morphometric analyses were performed using ImageJ software. Quantitative measurements focused on muscle fiber cross-sectional area as an indicator of fiber atrophy. Values were also used to evaluate the fiber size distribution and grouped into four categories, considering that the average cross-sectional area of the different fiber types is normally distributed as follows:

- Type I fibers: 300–1300 µm^2^
- Type IIa fibers: 1300–3000 µm^2^
- Type IIx fibers: 3000–4800 µm^2^
- Type IIb fibers: >4800 µm^2^

Although not based on specific molecular markers or metabolic properties, this morphological classification provides a reasonable estimate of the muscle fiber populations preferentially affected or preserved during disease progression and in response to treatment. Centrally located nuclei (CLN) were quantified using ImageJ, normalized to the total number of fibers, and expressed relative to WT values.

In order to evaluate the muscle innervation and its modification due to disease progression or treatments, other series of sections from the same subjects were immunostained to analyze acetylcholinesterase (AChE) expression in the cholinergic terminals at NMJs. In more details, sections were rehydrated and endogenous peroxidase activity was quenched by 3% hydrogen peroxide in PBS; then, sections were incubated overnight with the rabbit polyclonal anti-AChE primary antibody (Proteintech, Manchester, UK; Cat# 17975-1-AP, RRID: AB_2242349; 1:50). After repeated rinses, sections were then incubated with a universal streptavidin-HRP system (LSAB2 System-HRP, DakoCytomation, Carpinteria, CA, USA; Cat# K0690) and then reacted with 0.025% diaminobenzidine (DAB; Sigma) and 0.1% hydrogen peroxide in PBS for 3 min. Sections were then dehydrated, coverslipped and images were acquired by optical microscope and analyzed with ImageJ. AChE expression was evaluated by semi-quantitative image analysis according to the protocol described by Crowe and Yue [35]. Images were subjected to color deconvolution in ImageJ to isolate the specific DAB signal. A standardized threshold was then applied to the DAB channel to remove background staining, and the area occupied by the positive signal was measured as a quantitative index of AChE expression.

### 2.5. Western Blot Analysis

Isolated spinal cord tissue was lysed in RIPA buffer supplemented with protease and phosphatase inhibitors. Samples were mechanically homogenized until a homogeneous lysate was obtained and subsequently centrifuged at 12,000 rpm for 30 min at 4 °C using a refrigerated centrifuge. Following centrifugation, the cleared supernatant was collected and transferred to fresh tubes for analyses. Protein concentration was determined by bicinchoninic acid (BCA) assay. Equal amounts of protein from each sample were mixed with 5× loading buffer and denatured at 100 °C for 5 min prior to electrophoresis. Proteins were separated on 4–20% SDS–polyacrylamide gels prepared in a Tris–glycine buffer system and then transferred onto nitrocellulose membranes. Each sample was run at least in triplicate.

Membranes were blocked for 1 h at RT in 5% BSA and subsequently incubated for 2 h with the primary antibody diluted in 1% BSA in Tris-buffered saline containing 0.1% Tween-20 (TBST). The following primary antibodies were used: rabbit monoclonal anti-Drp-1 (D6C7) (Cell Signaling Technology, Danvers, MA, USA; Cat# 8570, RRID: AB_10950498; 1:500), rabbit polyclonal anti-Synapsin-1 (Proteintech, Rosemont, IL, USA; Cat# 20258-1-AP, RRID: AB_2800493; 1:1000) and mouse monoclonal anti-β-actin (Proteintech, Rosemont, IL, USA; Cat# 66009-1-Ig, RRID: AB_2687938; 1:15,000). After primary antibody incubation, membranes were washed three times in TBST for 5 min each and then incubated for 1 h at room temperature with HRP-conjugated secondary antibodies (goat anti-rabbit IgG-HRP, Invitrogen, Cat# 31460, RRID: AB_228341, 1:15,000; goat anti-mouse IgG-HRP, Invitrogen, Cat# 31430, RRID: AB_228307, 1:20,000) diluted in 2% BSA in TBST. Protein immunoreactivity was detected using an enhanced chemiluminescence HRP substrate. Images were acquired at least in duplicate (different exposition) by Uvitec Alliance LD2 (Cambridge, UK), and band intensities were quantified using ImageJ software.

### 2.6. Statistical Analysis

Sample size was established through power analysis using the rotarod data as the primary outcome measure. Data distribution was assessed for normality using the D’Agostino and Pearson omnibus normality test, followed by evaluation of variance homogeneity. Results are presented as mean ± SEM (standard error of the mean) and, when appropriate, normalized to control values. Statistical differences between experimental groups were analyzed using one-way or two-way ANOVA followed by either Tukey’s or Dunnett’s post hoc tests, as appropriate for the dataset. Statistical significance was set at *p* < 0.05 for all analyses. Data processing and statistical analyses were carried out using Systat software version 11 (Systat Software, Inc., Chicago, IL, USA) and GraphPad Prism (version 5.01).

## 3. Results

### 3.1. Mdivi-1 Treatment Causes Decline in Motor Performance

**Figure 1:**
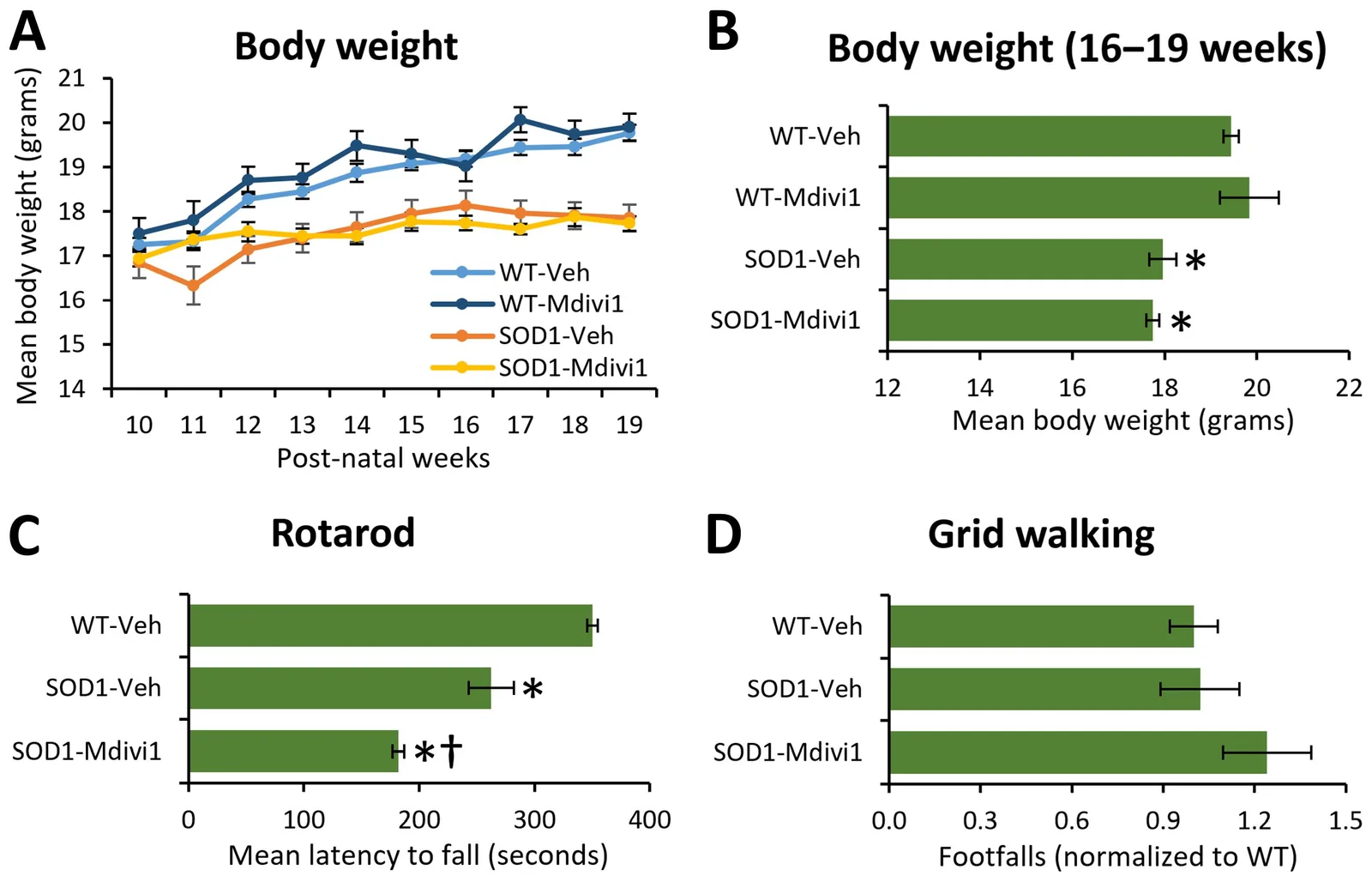
Body weight and motor performance in WT and SOD1 mice treated with either Mdivi-1 or vehicle alone (WT-Veh, *n* = 17; WT-Mdivi1, *n* = 5; SOD1-Veh, *n* = 13; SOD1-Mdivi1, *n* = 17). Longitudinal (**A**) and endpoint (**B**) body weight analysis confirm drug tolerability without evidence of systemic toxicity, and a significant reduction in body weight in all SOD1 mice, irrespective of the treatment. Rotarod (**C**) test shows a reduction in performance in SOD1 animals and a detrimental effect of Mdivi-1 treatment. Grid walk tests (**D**) show no modifications of motor coordination. The performance was assessed by counting the number of footfalls in each trial. Asterisks indicate statistically significant difference from WT levels, while the dagger (†) denotes significant difference from the SOD1-Veh group.

To monitor disease progression and assess potential systemic toxicity or adverse effects of Mdivi-1, body weight was measured weekly from the 10th post-natal week until sacrifice. Drug administration started at 12 post-natal weeks and continued until the end of the study. Longitudinal analysis of body weight progression (Figure 1A) revealed that SOD1^G93A^ mice displayed a slower weight gain compared with WT animals, consistent with disease progression.

In particular, two-way ANOVA revealed significant difference among groups (*p* < 0.0001), weeks (*p* < 0.0001) and group × week interaction (*p* = 0.048), suggesting that the longitudinal variation in body weight diverged across groups. In fact, the difference among groups was statistically significant from week 12 onwards (One-way ANOVA, *p* < 0.01; Tukey’s post hoc test, *p* < 0.05; Figure 1A). At the end of experimental period (16–19 weeks), the mean body weight was found reduced by about 10% in SOD1 mice treated with either vehicle or Mdivi-1, compared to either WT-Veh or WT-Mdivi1 mice (One-way ANOVA, *p* < 0.0001; Tukey’s post hoc test, *p* < 0.001; Figure 1A,B). No differences were observed between Mdivi-1-treated WT and SOD1 mice (Tukey’s post hoc test, *p* > 0.05) as well as between SOD1-Mdivi1 and SOD1-Veh mice (Tukey’s post hoc test, *p* > 0.05), indicating that the compound did not significantly affect general energy balance or growth trajectories. Given these results, WT-Mdivi1 mice were not included in the motor tests and subsequent post-mortem analyses.

Motor behavior was assessed weekly from the 13th to the 19th post-natal week using the rotarod and grid walking tests. For each animal, the mean performance over this interval was computed and then averaged across the respective experimental group (Figure 1C,D). As expected, SOD1-Veh mice showed a reduced (by about 25%) latency to fall at the rotarod test compared to WT mice (One-way ANOVA, *p* < 0.001; Tukey’s post hoc test, *p* = 0.006; Figure 1C), whereas no significant difference was found in grid walking performance (One-way ANOVA, *p* > 0.05; Figure 1D). Unexpectedly, Mdivi-1 treatment caused an additional 30% decrease in rotarod performance, compared to the SOD1-Veh mice (Tukey’s post hoc test, *p* = 0.007; Figure 1C) and a slight increase in the number of footfalls at the grid walk test, which is consistent with the pattern observed in the rotarod test, suggesting a subtle worsening of fine motor control that did not reach statistical significance (One-way ANOVA, *p* > 0.05; Figure 1D). Collectively, these findings indicate that, while Mdivi-1 is well tolerated, it does not mitigate motor deficits in this aggressive ALS model and may, under certain conditions, negatively affect coordination and balance in SOD1^G93A^ mice.

### 3.2. Spinal MN Survival

**Figure 2:**
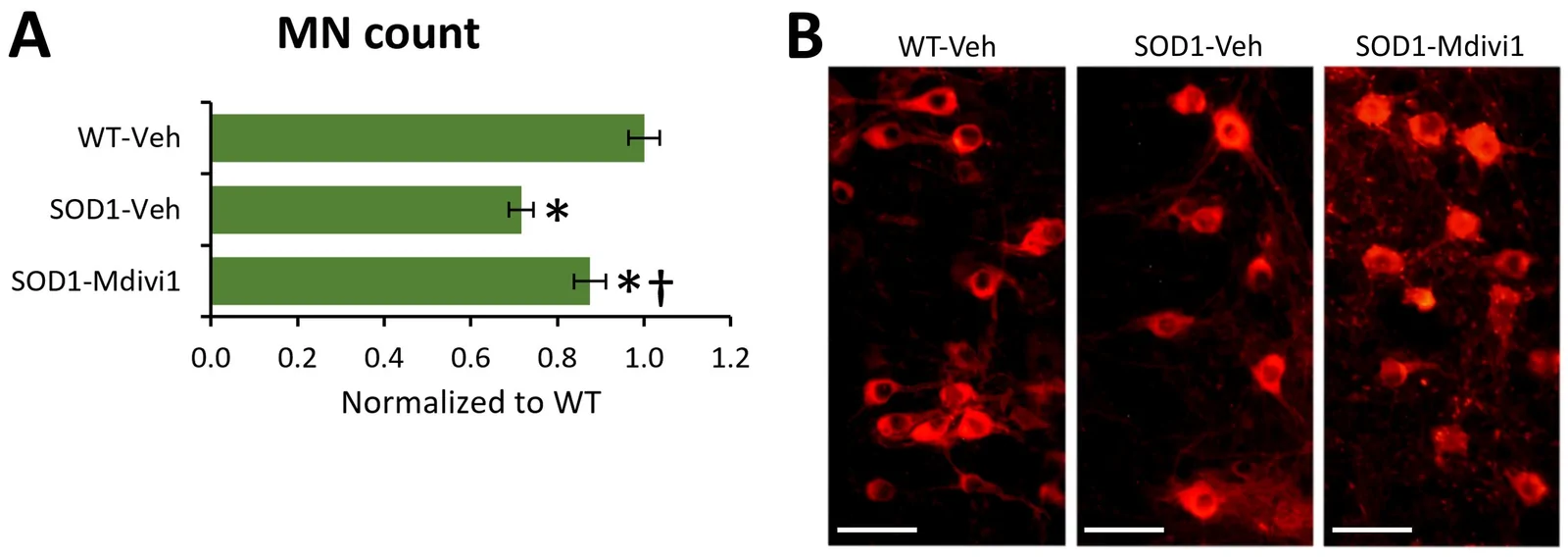
Counting (**A**) of ChAT-positive (red) MNs in immunostained spinal cord sections (**B**). An average 30% reduction in MN-like cells was observed in SOD1 animals treated with vehicle (**A**), while a partial MN sparing was observed after Mdivi-1 treatment. Asterisks indicate statistically significant difference from WT levels, while the dagger (†) denotes significant difference from the SOD1-Veh group. Analyses were conducted on 4 sections/animal (WT-Veh, *n* = 4 mice; SOD1-Veh, *n* = 3 mice; SOD1-Mdivi1, *n* = 4 mice). Scale bars: 50 μm.

MN integrity in the spinal cord was evaluated using ChAT immunofluorescence, allowing for selective visualization and quantification of cholinergic MNs (Figure 2B). Quantitative analysis of spinal MN-like cells revealed that Mdivi-1 treatment exerted a measurable protective effect in SOD1^G93A^ mice. Immunofluorescence showed a pronounced 28% reduction in ChAT-positive MN-like somata in SOD1-Veh animals (One-way ANOVA, *p* < 0.001; Tukey’s post hoc test, *p* < 0.001; Figure 2A,B), consistent with the typical degeneration occurring in this animal model. In contrast, Mdivi-1-treated SOD1 mice displayed a significantly higher number of ChAT-positive MN-like cells within the spinal cord, compared to SOD1-Veh mice (Tukey’s post hoc test, *p* = 0.002; Figure 2A,B), indicating a partial rescue of MN degeneration (only a 13% reduction from WT; Tukey’s post hoc test, *p* = 0.038). Interestingly, this structural preservation occurred even in the absence of measurable improvements in motor performance. This dissociation suggests that Mdivi-1 may exert neuroprotective actions at the cellular level that precede, but does not necessarily guarantee functional rescue in later disease stages.

### 3.3. TDP-43 Subcellular Localization in Spinal MNs

**Figure 3:**
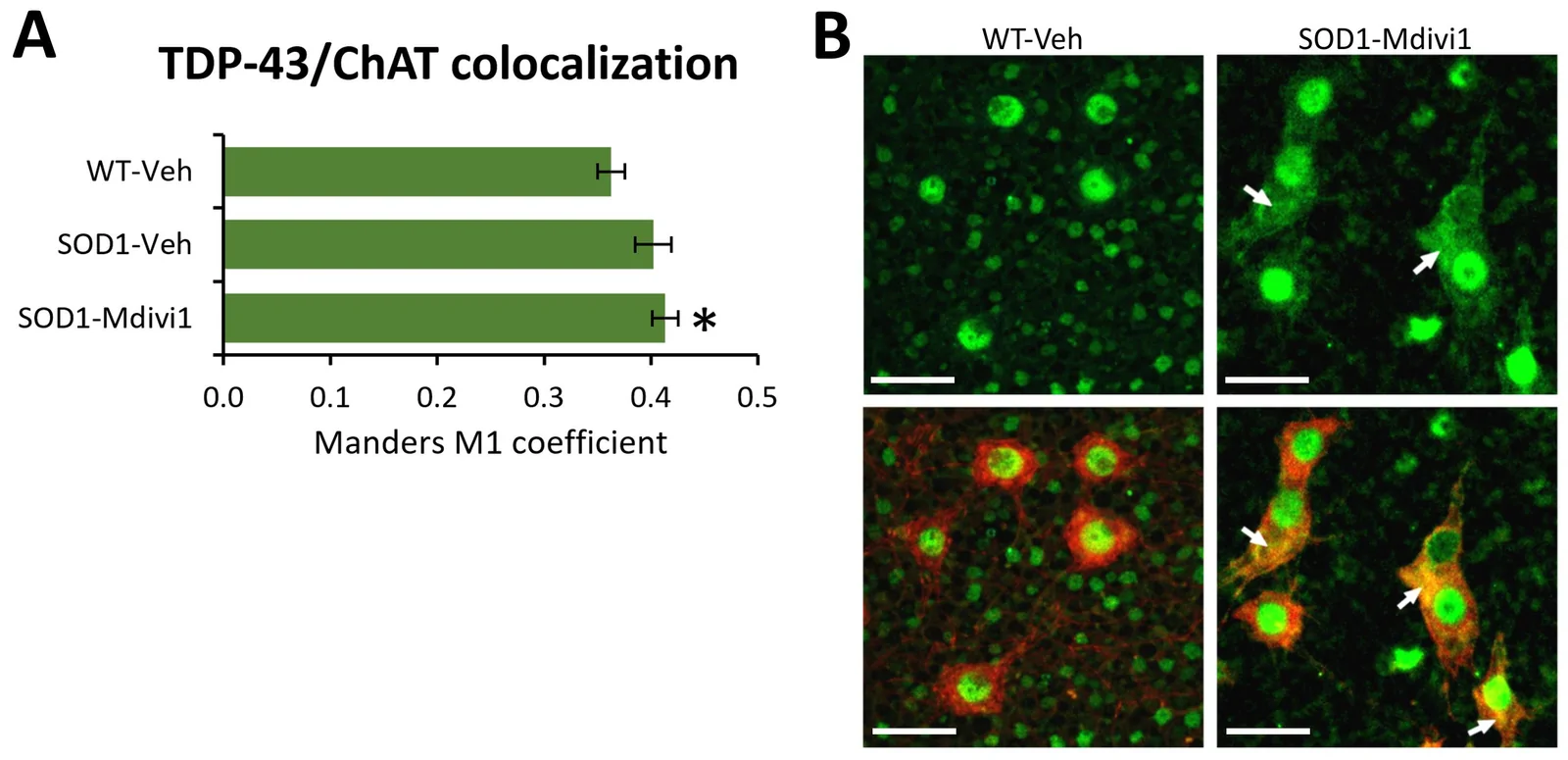
Expression of TDP-43 in the spinal cord of WT and SOD1 mice, as assessed by immunofluorescence. In particular, the graph in (**A**) reports the amount of colocalization (Manders’ M1 coefficient, please see Methods, Section 2.4) of TDP-43 (green signal in (**B**)) and ChAT (red) in spinal MNs. The asterisk indicates statistically significant difference from WT-veh group. Arrows in (**B**) indicate the most evident areas of colocalization in SOD1 mice treated with Mdivi-1, suggesting cytoplasmic mislocalization of TDP-43. Analyses were conducted on 4 sections/animal (WT-Veh, *n* = 4 mice; SOD1-Veh, *n* = 3 mice; SOD1-Mdivi1, *n* = 4 mice). Scale bars: 30 μm.

To further investigate the pathological state of surviving MNs, we next examined the subcellular localization of TDP-43 by double immunofluorescence staining for ChAT and TDP-43 in the spinal cord (Figure 3). TDP-43 is an RNA-binding protein that, in ALS and related neurodegenerative conditions, displays abnormal cytoplasmic accumulation and aggregation, reflecting impaired cellular proteostasis. Therefore, its distribution provides important insight into disease-associated cellular dysfunction beyond neuronal survival.

Analysis of spinal cord sections revealed an increase in cytoplasmic TDP-43 immunoreactivity in SOD1^G93A^ mice compared to WT controls, consistent with the presence of TDP-43 pathology in this model. Quantification of cytoplasmic TDP-43 accumulation within ChAT-positive MNs was performed using Manders’ M1 colocalization coefficient. Although SOD1-Veh mice showed a trend toward increased TDP-43/ChAT cytoplasmic colocalization relative to WT animals (+11%), this difference did not reach statistical significance (One-way ANOVA, *p* = 0.049; Dunnett’s post hoc test, *p* > 0.05; Figure 3A). Conversely, Mdivi-1 treatment caused a higher increase in cytoplasmic TDP-43 colocalization compared to WT controls (+15%; Dunnett’s post hoc test, *p* < 0.05; Figure 3A,B). These findings indicate that, despite promoting partial preservation of spinal MNs, modulation of mitochondrial fission does not prevent, and may even be associated with a persistence or slight exacerbation of TDP-43 pathology. Overall, these results suggest that mitochondrial dysfunction in ALS is complex and may differentially affect distinct pathological pathways in ALS.

### 3.4. Muscle Atrophy and Regenerative Response

**Figure 4:**
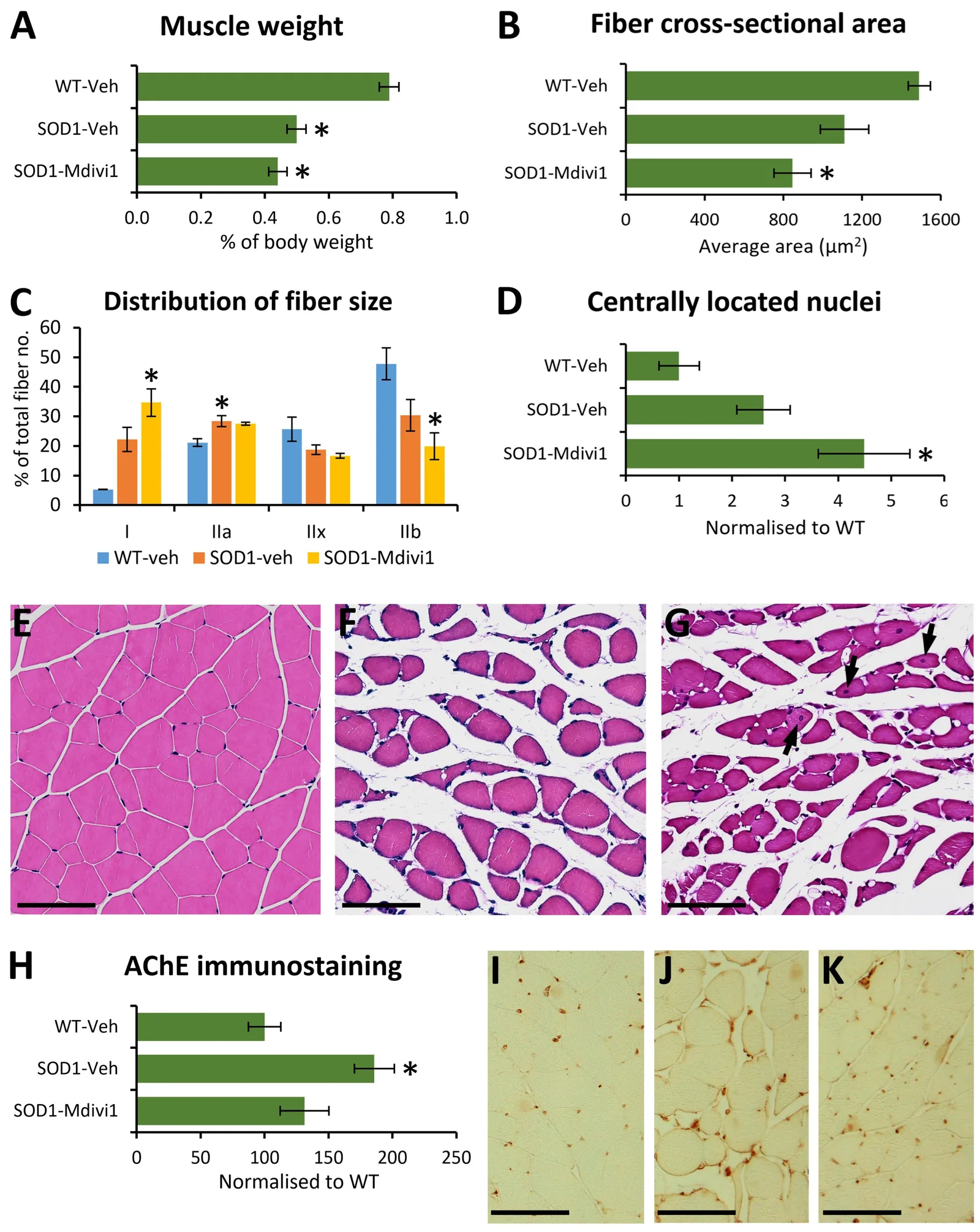
Histological (H&E) and immunohistochemical analyses of gastrocnemius muscle and estimation of muscle atrophy and cholinergic innervation. Morphology and atrophy were assessed by measuring the muscle weight (**A**) and the mean cross-sectional area of muscle fibers (**B**,**E**–**G**) in WT-Veh (**E**), SOD1-Veh (**F**) and SOD1-Mdivi1 (**G**) mice. Gastrocnemius muscle weight has been normalized to body weight to account for the effects of disease progression and drug treatment. Fiber cross-sectional area was also used to estimate the distribution of fiber type I, IIa, IIx and IIb based on morphological criteria (**C**). The number of CLN (indicated by arrows in (**G**)) relative to the total number of fibers in the muscle sections were normalized to WT values (**D**,**E**–**G**). The presence of CLN can be interpreted as a sign, although insufficient, of an attempted regeneration of muscle tissue. The innervation of muscle fibers by cholinergic motor neurons has been assessed by AChE immunostaining (**H**–**K**). The graph in (**H**) shows the optical density of immunostaining (please see Methods, Section 2.4 for details), normalized to WT levels, in WT-Veh (**I**), SOD1-Veh (**J**) and SOD1-Mdivi1 (**K**) mice. Asterisks indicate a statistically significant difference from the WT controls. Analyses were conducted on 3 sections/animal (WT-Veh, *n* = 4 mice; SOD1-Veh, *n* = 3 mice; SOD1-Mdivi1, *n* = 4 mice). Scale bars: 100 μm.

Muscle atrophy was evaluated by measuring gastrocnemius weight normalized to body weight, the mean fiber cross-sectional area, and fiber size distribution, as described in the Methods, Section 2.4 (Figure 4).

Gastrocnemius weight was significantly reduced in SOD1-Veh mice compared to WT controls (by about 37%; One-way ANOVA, *p* < 0.001; Tukey’s post hoc test, *p* < 0.001; Figure 4A), consistent with the progressive muscle wasting characteristic of this model. The mean cross-sectional area of muscle fibers confirmed this atrophic pattern, revealing an evident 27% reduction in SOD1-Veh animals relative to WT controls, although it did not reach statistical significance (One-way ANOVA, *p* < 0.01; Tukey’s post hoc test, *p* = 0.068; Figure 4B,E–G). SOD1-Mdivi1 mice showed a similar reduction in muscle weight but a lower mean fiber cross-sectional area, indicating an accentuation of the atrophic phenotype in treated animals (about 43% reduction from WT controls; One-way ANOVA, *p* < 0.01; Tukey’s post hoc test, *p* = 0.007; Figure 4B,E–G).

Fiber-type composition of the muscle was subsequently analyzed based on morphological appearance (cross-sectional area) to characterize the disease-associated remodeling of the muscle.

SOD1-Veh mice displayed an apparently higher (but not statistically significant) proportion of type I (One-way ANOVA, *p* = 0.014; Tukey’s post hoc test, *p* > 0.05; Figure 4C,E–G) and a higher proportion of type IIa (One-way ANOVA, *p* = 0.034; Tukey’s post hoc test, *p* < 0.05; Figure 4C,E–G) fibers and a reduction (although not statistically significant) in type IIb fibers (One-way ANOVA, *p* = 0.039; Tukey’s post hoc test, *p* > 0.05; Figure 4C,E–G) relative to WT controls, reflecting the typical fast-to-slow shift in fiber-type distribution associated with disease progression. This compositional shift was further accentuated in SOD1-Mdivi1 animals, which exhibited a greater proportion of type I fibers (Tukey’s post hoc test, *p* < 0.05 compared to WT; Figure 4C,E–G) and a more pronounced reduction in type IIb fibers (Tukey’s post hoc test, *p* < 0.05 compared to WT; Figure 4C,E–G). No significant differences in type IIx fiber proportion were observed across groups (One-way ANOVA, *p* = 0.071), despite an apparent reduction in both SOD1 groups. The number of myofibers with CLN was quantified as a histological indicator of ongoing myofiber regeneration. Mdivi-1-treated SOD1^G93A^ animals exhibited a significant increase in the proportion of fibers with CLN compared with WT controls (One-way ANOVA, *p* = 0.011; Tukey’s post hoc test, *p* = 0.009; Figure 4D,E–G), indicating an enhancement of myofiber regenerative activity in treated animals.

To assess muscle innervation, transverse sections of the gastrocnemius muscle were processed for immunohistochemistry using an anti-AChE antibody, which labels cholinergic synapses within the skeletal muscle. Optical density quantification revealed an approximately 85% increase in AChE immunostaining in SOD1-Veh mice compared with WT-Veh animals (One-way ANOVA, *p* = 0.012; Tukey’s post hoc test, *p* < 0.05; Figure 4H; compare Figure 4I with Figure 4J), whereas SOD1-Mdivi1 mice showed values comparable to those of the control group (Tukey’s post hoc test, *p* > 0.05; Figure 4H; compare Figure 4I with Figure 4K).

### 3.5. Western Blot Analysis of Drp-1 and Synapsin-1 Protein Expression

To examine whether Mdivi-1 treatment influenced mitochondrial fission signaling, Drp-1 protein expression was assessed in spinal cord tissue extracts by Western blot.

**Figure 5:**
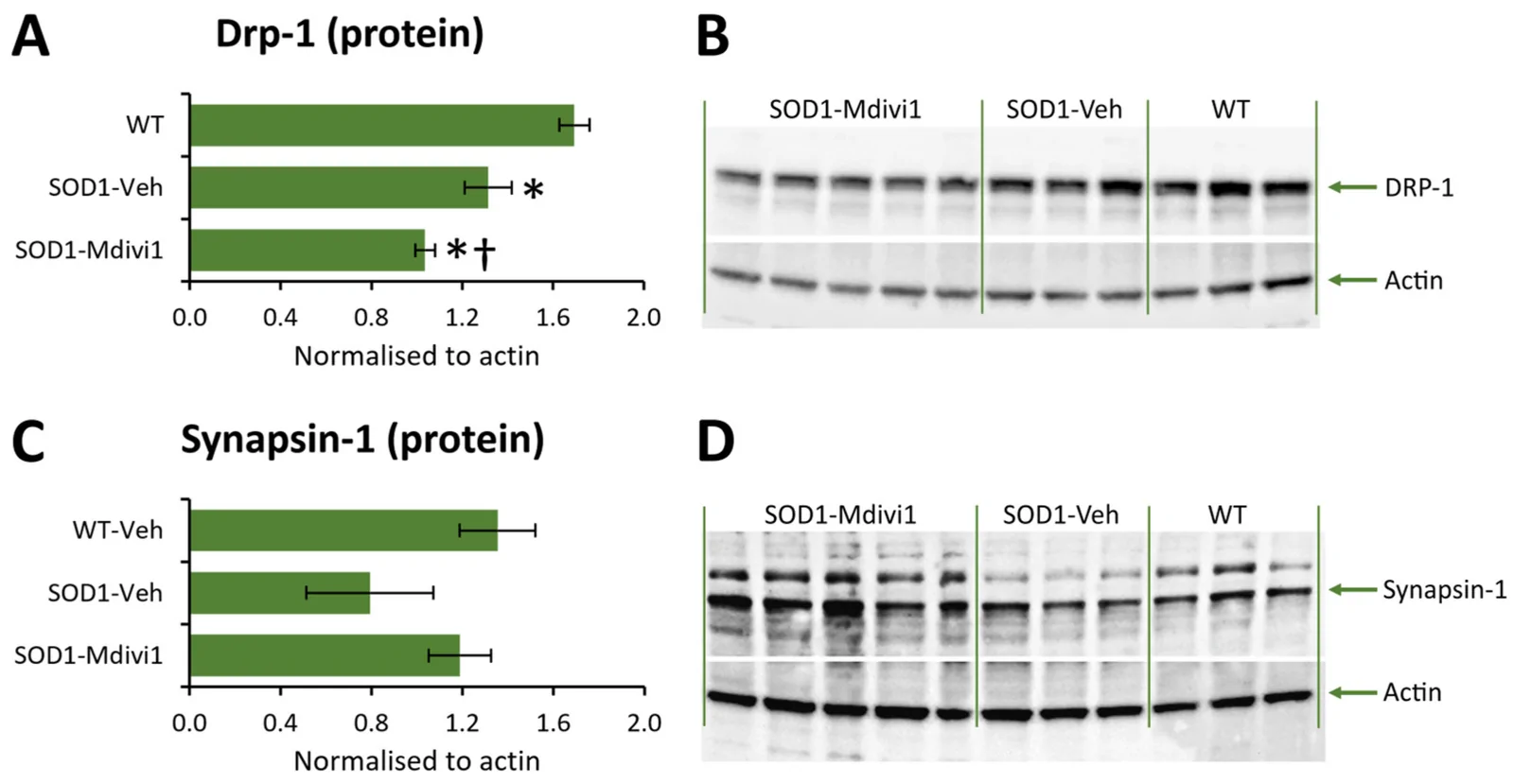
Expression of Drp-1 and Synapsin-1 proteins in the spinal cord of WT (*n* = 7) and SOD1 mice treated with Mdivi-1 (*n* = 5) or vehicle alone (*n* = 5), as assessed by Western blot, at least in triplicate. In particular, the graphs in (**A**) and (**C**) show the mean protein levels normalized to actin and expressed as arbitrary units of optical density, while the pictures in (**B**) and (**D**) represent examples of the relative gels. Asterisks denote statistically significant difference from the WT group, whereas † indicates a significant difference from the SOD1-Veh group. Uncropped images can be found in Supplementary Materials.

Samples were collected from SOD1-Veh mice (*n* = 5), SOD1-Mdivi1 (*n* = 5), and age-matched WT controls (*n* = 7). Given the extensive literature linking Drp-1-driven mitochondrial fragmentation to neuronal dysfunction and degeneration, we hypothesized that Drp-1 expression would be upregulated in SOD1^G93A^ mice. Contrary to this prediction, densitometric quantification revealed reduced Drp-1 protein levels in untreated SOD1 mice relative to WT controls (One-way ANOVA, *p* < 0.001; Tukey’s post hoc test, *p* = 0.032; Figure 5A,B), and a further downregulation in Mdivi-1-treated animals (Tukey’s post hoc test, *p* = 0.001 compared to WT, *p* = 0.046 compared to SOD1-Veh; Figure 5A,B; uncropped images can be found in Supplementary Materials).

Synapsin-1 is a synaptic vesicle protein involved in the turnover of synaptic vesicles between the reserve pool and the readily releasable pool, and its expression is generally linked to synaptic plasticity. Here, the expression of this protein in the spinal cord appeared reduced in SOD1-Veh and similar to WT controls in the Mdivi-1-treated mice, but these modifications were not statistically significant (One-way ANOVA, *p* = 0.157; Figure 5C,D; uncropped images can be found in Supplementary Materials).

## 4. Discussion

Mdivi-1, a cell-permeable quinazolinone, has been proposed as a potential therapeutic candidate for neurodegenerative diseases, largely due to its reported ability to modulate mitochondrial dynamics by inhibiting the mitochondrial fission protein Drp-1 [36]. However, studies in models of neurodegeneration and acute injury not only show that Mdivi-1 reduces mitochondrial fragmentation, but that it can also attenuate oxidative stress, preserve mitochondrial membrane potential, sustain synaptic maintenance and transmission, and limit apoptosis, thereby improving cellular survival and tissue integrity [16,17,37,38,39,40].

The present study has investigated the effects of Mdivi-1 administration in the SOD1^G93A^ mouse model, focusing on both central and peripheral components of the neuromuscular system. Mdivi-1 treatment significantly preserved spinal MN integrity, yet failed to improve motor performance and was associated with worsening muscle pathology and alterations in protein homeostasis, such as increased cytoplasmic TDP-43 localization. However, no signs of general drug toxicity were found that can justify such functional detrimental effects. These findings challenge the assumption that inhibition of mitochondrial fission is uniformly beneficial in neurodegenerative conditions and suggest that its effects may be highly context-dependent. This work was directly motivated by previous findings from our laboratory showing that Mdivi-1 effectively mitigates functional deficits following selective MN removal induced by intramuscular CTB-Sap injection [27]. In contrast to that model characterized by rapid and spatially restricted neuronal loss, the SOD1^G93A^ model recapitulates a progressive and multifactorial pathology affecting multiple tissues and cellular pathways. This divergence likely reflects the complex nature of ALS, in which pathological processes evolve across different tissues and disease stages [33,41,42]. In particular, it is important to note that the CTB-Sap neurotoxic model retains a remarkable degree of spontaneous (but also targetable by treatments) plasticity following neuronal loss, being able to recover, at least partially, motor function over the course of several weeks after the lesion [27,43,44,45,46]. A similar plastic capacity is also present in the SOD1 mouse model, but probably more efficiently during the very early stages of the disease, where it is able to functionally compensate for motor neuron loss, which is already evident at early stages and progressively worsens over time. Indeed, the expression of Synapsin-1, a protein widely associated with synaptic plasticity [47,48], suggests that plastic remodeling within the spinal cord circuitry is likely insufficient to counteract the effects of disease progression. Specifically, Synapsin-1 expression was moderately reduced in SOD1-Veh animals and partially restored following Mdivi-1 treatment. This pattern (only a trend of modification) is likely to reflect the preservation of motor neurons rather than an enhancement of synaptic connectivity or strength, as no evidence of a treatment-induced increase beyond control levels was observed. Most likely, it is the failure of these plastic compensatory mechanisms, together with the progressive loss of motor neurons, that ultimately leads to symptom onset [49,50,51,52]. This suggests that, by the time onset approaches, compensatory processes are already under considerable strain and are therefore less capable of responding to pharmacological intervention. Indeed, the timing of pharmacological interventions in SOD1 mouse models has emerged as a crucial factor influencing both disease onset and progression [53]. Future studies employing treatment paradigms initiated at different stages of the disease may yield valuable insights not only into the translational potential of Mdivi-1-based strategies, but also into the sequence of pathogenic events that collectively drive ALS onset and progression [10,53,54].

Mitochondrial dynamics are increasingly recognized as part of a tightly regulated stress response, allowing cells to maintain bioenergetic flexibility, remove damaged organelles, and adapt to metabolic demands [18,55,56]. Although excessive mitochondrial fission has been widely implicated in neurodegenerative pathology, emerging evidence suggests that components of the fission machinery may also support adaptive stress responses in chronically degenerating neurons [29], so its pharmacological inhibition could likely disrupt cellular homeostasis rather than restore it [28]. This interpretation is supported by a recent study demonstrating that Mdivi-1 administration following status epilepticus exacerbates microglia-mediated neuroinflammation and impairs brain glucose metabolism [57]. So, the modulation of mitochondrial dynamics can have unexpected consequences on non-neuronal cells and metabolic networks, ultimately worsening disease-related processes. In line with these observations, the absence of functional improvement in our study, despite partial MN preservation, may reflect a broader disruption of cellular and metabolic homeostasis, involving also other neural cells and muscle. In any case, although Mdivi-1 treatment increased the number of surviving ChAT-positive MNs, preservation of neuronal somata does not necessarily imply maintenance of their physiological competence. Several findings from the present experiments support this hypothesis. For example, the spinal expression of Synapsin-1, discussed above, did not show significant changes in response to either disease progression or treatment. This observation is complemented by the findings on skeletal muscle innervation, which appeared to be increased in SOD1-Veh mice, likely reflecting compensatory reinnervation processes that are well-documented in the SOD1 model [50]. During the early and intermediate stages of the disease, these compensatory mechanisms are thought to partially offset the progressive loss of motor neurons. In contrast, this compensatory response appeared to be attenuated in Mdivi-1-treated animals. Taken together, these findings are consistent with the possibility that, although MNs were partially preserved following Mdivi-1 treatment, they may have remained functionally compromised and are therefore less able to sustain effective spinal circuit integration and neuromuscular connectivity. However, this interpretation should be considered as one possible explanation that warrants further investigation, such as a more direct assessment of MN activity, or deeper investigation of NMJ morphology and muscle innervation. ALS pathology affects the entire motor unit, including axonal transport, neuromuscular junction stability, synaptic connectivity, and skeletal muscle integrity, all of which contribute to motor performance and are altered during the progression of disease [49,50,51,52,58,59,60]. Thus, neurons may remain morphologically identifiable while exhibiting substantial molecular and functional abnormalities. Together with the above-described expression of Synapsin-1 and AChE, the increased cytoplasmic localization of TDP-43 observed in MNs of Mdivi-1-treated mice may indicate that surviving MNs remain under considerable cellular stress [61]. It should be noted, however, that the present analysis provides only an indirect estimate of cytoplasmic TDP-43 localization, based on colocalization with the cholinergic marker ChAT, and does not assess the nuclear-to-cytoplasmic TDP-43 ratio. TDP-43 mislocalization derives from multiple pathogenic mechanisms, but it is also considered an early marker of neuronal dysfunction and impaired RNA metabolism rather than a direct indicator of imminent cell death [62,63]. Consequently, Mdivi-1 may delay neuronal loss, at least until our examined endpoint, while failing to correct the pathological mechanisms responsible for neuronal dysfunction. Such a scenario would explain how partial (and probably transient) neuroprotection can coexist with persistent or even aggravated molecular pathology. Similar dissociations between neuronal survival and functional outcome have been reported in several experimental models of neurodegeneration, where interventions preserve cell numbers without restoring network integrity or behavioral performance [58,59].

Another finding of this study is the reduction in Drp-1 protein levels in SOD1^G93A^ mice, which was further decreased following Mdivi-1 treatment. This result contrasts with the commonly assumed upregulation of Drp-1 in pathological conditions [21,64,65] and may be consistent with a progressive alteration in mitochondrial fission capacity during disease progression, although the present study did not directly assess Drp-1 activity or mitochondrial dynamics. Because Drp1 plays an important role in mitochondrial quality control, the reduction in Drp1 protein observed in the present study raises the possibility that these processes may also be compromised [66]. Therefore, reduced Drp-1 levels may impair the ability of cells to selectively eliminate dysfunctional mitochondria, leading to the accumulation of bioenergetically compromised organelles. In this context, further reduction in Drp-1 protein levels may not simply reduce fission but may actively hinder mitochondrial quality control mechanisms, exacerbating cellular stress and also probably impairing synaptic maintenance and transmission. However, this hypothesis will require direct evaluation of mitochondrial dynamics, mitophagy and Drp1 activation state in future studies.

The exacerbation of the typical ALS-linked muscle atrophy observed in SOD1 animals treated with Mdivi-1 further confirms this complex pathological context. In our previous study using a model of selective MN loss [27], Mdivi-1 was associated with improved motor performance and the preservation of muscle integrity, consistent with a role for mitochondrial dynamics in supporting adaptive responses to denervation. In this setting, skeletal muscle underwent a rapid and focal denervation in the absence of pre-existing pathology, thereby maintaining sufficient metabolic flexibility to engage compensatory and regenerative mechanisms. In contrast, accumulating evidence indicates that, in ALS, skeletal muscle is not merely a passive target of MN degeneration but may represent an early and actively involved compartment in disease pathogenesis [67]. Alterations in mitochondrial dynamics have been reported in skeletal muscle following the expression of mutant SOD1^G93A^ even in the absence of overt MN loss, indicating that the mutation itself is sufficient to drive muscle pathology [30]. Our data show marked muscle fiber degeneration and exacerbated atrophy in SOD1^G93A^ mice treated with Mdivi-1, suggesting that the muscle compartment may already be severely compromised at the time of intervention and particularly vulnerable to further metabolic perturbations. Therefore, the pronounced fiber-size remodeling observed in Mdivi-1-treated mice, together with the observed increase in CLN, likely reflects an acceleration of the denervation–reinnervation cycle that characterizes ALS pathology. In experimental models, neuromuscular junction destabilization and distal axonal withdrawal often precede overt MN loss, suggesting that pathological processes may initially emerge at the peripheral level of the motor unit [58,59]. The reduced muscle innervation observed in Mdivi-1-treated animals, despite the partial preservation of motor neurons, may suggest a peripheral effect of the compound. However, it is also consistent with the possibility that the surviving motor neurons were functionally impaired, despite remaining structurally preserved.

In skeletal muscle, sustained oxidative stress may further compromise mitochondrial function and regenerative capacity, contributing to the observed shift toward smaller fiber types and increased number of fibers with CLN. While these features are indicative of an attempted regenerative response, they appear insufficient to counterbalance ongoing degeneration [68]. Importantly, the increase in CLN number should not necessarily be interpreted as evidence of successful regeneration. Rather, the elevated proportion of CLN-positive fibers may reflect an increased requirement for regenerative activity secondary to greater tissue injury. It is possible that extending the experimental period, and treatment duration, would have revealed more evident signs of regeneration, despite the possibly increased severity of disease. In chronic neuromuscular disorders, regeneration markers frequently increase in parallel with disease severity because repeated cycles of degeneration and regeneration are continuously activated but fail to fully restore tissue integrity [68,69]. Accordingly, the increased number of CLN observed following Mdivi-1 treatment may represent a compensatory response to exacerbated muscle damage rather than an indication of improved muscle recovery.

Moreover, recent evidence suggests that the actions of Mdivi-1 may extend beyond the inhibition of Drp-1-mediated fission. In particular, a detailed analysis of its impact on mitochondrial bioenergetics has shown that Mdivi-1 can directly influence complex I activity and the assembly of respiratory complexes, thereby altering electron transport chain efficiency and overall mitochondrial respiration [29]. These findings indicate that, in addition to its role in Drp-1 inhibition, Mdivi-1 may exert broader effects on mitochondrial function that are not only related to fission dynamics. Notably, in metabolically fragile systems such as ALS, where mitochondrial bioenergetic capacity is already compromised [70], even subtle perturbations of respiratory efficiency may have deleterious consequences. Taken together, these observations suggest that mitochondrial fission should not be regarded solely as a pathogenic mechanism in ALS. Rather, under conditions of chronic cellular stress, impaired bioenergetics, and defective proteostasis, mitochondrial fission may also contribute to adaptive responses aimed at preserving organelle quality control and metabolic flexibility. In addition, Drp1-mediated mitochondrial fission is required for the proper redistribution of mitochondria along axons and dendrites, ensuring their delivery to regions of high metabolic demand, including synaptic terminals, where local ATP production is essential for synaptic transmission and maintenance [16,17]. Therefore, the inhibition of Drp1 may compromise neuronal function not only by altering mitochondrial morphology, but also by impairing mitochondrial positioning and local energy supply within neuronal processes. Taken together, our findings are consistent with the hypothesis that preserving a certain level of Drp1 function may be important during symptomatic stages of ALS. This interpretation may help explain why Mdivi-1 produced partial structural neuroprotection while simultaneously aggravating several pathological features at both neuronal and muscular levels. Nevertheless, because the present study did not directly evaluate Drp1 activity or mitochondrial dynamics, this interpretation should be considered speculative and requires further experimental validation.

## 5. Conclusions

Collectively, these findings show that Mdivi-1 fails to restore cellular homeostasis and may instead reduce the capacity for metabolic adaptation across both central and peripheral compartments. The partial preservation of spinal MNs in the absence of functional benefit, and in the presence of exacerbated muscle pathology and TDP-43 mislocalization, together with impaired plasticity and muscle reinnervation, reveals a dissociation between neuronal survival and neuromuscular function that cannot be resolved by targeting mitochondrial fission alone. Future studies should therefore address whether earlier or more selective modulation of Drp-1 activity, integrated with complementary strategies targeting oxidative stress, proteostasis, or neuromuscular connectivity, may achieve a more effective neuroprotective outcome in this disease context.

## Data Availability

The data presented in this study are available on request from the corresponding author.

## Supplementary Materials

The following supporting information can be downloaded at https://www.mdpi.com/article/10.3390/biology15161334/s1, original uncropped Western blot images of Drp-1, Synapsin-1 and Actin protein levels.

## Abbreviations

The following abbreviations are used in this manuscript:

AChE	Acetylcholinesterase
ALS	Amyotrophic Lateral Sclerosis
BCA	Bicinchoninic Acid
ChAT	Choline Acetyltransferase
CLN	Centrally Located Nuclei
C9orf72	Chromosome 9 Open Reading Frame 72
CTB-Sap	Cholera Toxin B-subunit conjugated to Saporin
DAB	Diaminobenzidine
DMSO	Dimethyl Sulfoxide
Drp-1	Dynamin-related protein 1
Mdivi-1	Mitochondrial division inhibitor 1
MN	Motor Neuron
NDS	Normal Donkey Serum
OCT	Optimal Cutting Temperature
SOD1	Superoxide Dismutase 1
TDP-43	TAR DNA-binding protein 43

## References

[1] Klemmensen M.M., Borrowman S.H., Pearce C., Pyles B., Chandra B. (2024). Mitochondrial Dysfunction in Neurodegenerative Disorders. Neurotherapeutics.

[2] Su B., Wang X., Zheng L., Perry G., Smith M.A., Zhu X. (2010). Abnormal Mitochondrial Dynamics and Neurodegenerative Diseases. Biochim. Biophys. Acta.

[3] Sasaki S., Iwata M. (2007). Mitochondrial Alterations in the Spinal Cord of Patients with Sporadic Amyotrophic Lateral Sclerosis. J. Neuropathol. Exp. Neurol..

[4] Xiao Y., Karam C., Yi J., Zhang L., Li X., Yoon D., Wang H., Dhakal K., Ramlow P., Yu T. (2018). ROS-Related Mitochondrial Dysfunction in Skeletal Muscle of an ALS Mouse Model During the Disease Progression. Pharmacol. Res..

[5] Brown R.H., Al-Chalabi A. (2017). Amyotrophic Lateral Sclerosis. N. Engl. J. Med..

[6] Hardiman O., Al-Chalabi A., Chiò A., Corr E.M., Logroscino G., Robberecht W., Shaw P.J., Simmons Z., van den Berg L.H. (2017). Amyotrophic Lateral Sclerosis. Nat. Rev. Dis. Primers.

[7] Taylor J.P., Brown R.H., Cleveland D.W. (2016). Decoding ALS: From Genes to Mechanism. Nature.

[8] Renton A.E., Chiò A., Traynor B.J. (2014). State of Play in Amyotrophic Lateral Sclerosis Genetics. Nat. Neurosci..

[9] Kapeli K., Martinez F.J., Yeo G.W. (2017). Genetic Mutations in RNA-Binding Proteins and their Roles in ALS. Hum. Genet..

[10] Al-Chalabi A., Calvo A., Chiò A., Colville S., Ellis C.M., Hardiman O., Heverin M., Howard R.S., Huisman M.H.B., Keren N. (2014). Analysis of Amyotrophic Lateral Sclerosis as a Multistep Process: A Population-Based Modelling Study. Lancet Neurol..

[11] Magrané J., Hervias I., Henning M.S., Damiano M., Kawamata H., Manfredi G. (2009). Mutant SOD1 in Neuronal Mitochondria Causes Toxicity and Mitochondrial Dynamics Abnormalities. Hum. Mol. Genet..

[12] Stoica R., Paillusson S., Gomez-Suaga P., Mitchell J.C., Lau D.H., Gray E.H., Sancho R.M., Vizcay-Barrena G., De Vos K.J., Shaw C.E. (2016). ALS/FTD-Associated FUS Activates GSK-3β to Disrupt the VAPB—PTPIP 51 Interaction and ER–Mitochondria Associations. EMBO Rep..

[13] Wang W., Li L., Lin W.-L., Dickson D.W., Petrucelli L., Zhang T., Wang X. (2013). The ALS Disease-Associated Mutant TDP-43 Impairs Mitochondrial Dynamics and Function in Motor Neurons. Hum. Mol. Genet..

[14] Tilokani L., Nagashima S., Paupe V., Prudent J. (2018). Mitochondrial Dynamics: Overview of Molecular Mechanisms. Essays Biochem..

[15] Hu C., Huang Y., Li L. (2017). Drp1-Dependent Mitochondrial Fission Plays Critical Roles in Physiological and Pathological Progresses in Mammals. Int. J. Mol. Sci..

[16] Amiri M., Hollenbeck P.J. (2008). Mitochondrial Biogenesis in the Axons of Vertebrate Peripheral Neurons. Dev. Neurobiol..

[17] Ishihara N., Nomura M., Jofuku A., Kato H., Suzuki S.O., Masuda K., Otera H., Nakanishi Y., Nonaka I., Goto Y.-I. (2009). Mitochondrial Fission Factor Drp1 is Essential for Embryonic Development and Synapse Formation in Mice. Nat. Cell Biol..

[18] Youle R.J., van der Bliek A.M. (2012). Mitochondrial Fission, Fusion, and Stress. Science.

[19] Wang X., Su B., Lee H.-g., Li X., Perry G., Smith M.A., Zhu X. (2009). Impaired Balance of Mitochondrial Fission and Fusion in Alzheimer’s Disease. J. Neurosci..

[20] Iqbal S., Hood D.A. (2015). The Role of Mitochondrial Fusion and Fission in Skeletal Muscle Function and Dysfunction. Front. Biosci..

[21] Joshi A.U., Saw N.L., Vogel H., Cunnigham A.D., Shamloo M., Mochly-Rosen D. (2018). Inhibition of Drp1/Fis1 Interaction Slows Progression of Amyotrophic Lateral Sclerosis. EMBO Mol. Med..

[22] Rappold P.M., Cui M., Grima J.C., Fan R.Z., de Mesy-Bentley K.L., Chen L., Zhuang X., Bowers W.J., Tieu K. (2014). Drp1 Inhibition Attenuates Neurotoxicity and Dopamine Release Deficits in Vivo. Nat. Commun..

[23] Reddy P.H., Manczak M., Yin X. (2017). Mitochondria-Division Inhibitor 1 Protects Against Amyloid-β induced Mitochondrial Fragmentation and Synaptic Damage in Alzheimer’s Disease. J. Alzheimer’s Dis..

[24] Manczak M., Reddy P.H. (2015). Mitochondrial Division Inhibitor 1 Protects Against Mutant Huntingtin-Induced Abnormal Mitochondrial Dynamics and Neuronal Damage in Huntington’s Disease. Hum. Mol. Genet..

[25] Zhang Y., Rui T., Luo C., Li Q. (2021). Mdivi-1 Alleviates Brain Damage and Synaptic Dysfunction After Intracerebral Hemorrhage in Mice. Exp. Brain Res..

[26] Zong Y., Li H., Liao P., Chen L., Pan Y., Zheng Y., Zhang C., Liu D., Zheng M., Gao J. (2024). Mitochondrial Dysfunction: Mechanisms and Advances in Therapy. Signal Transduct. Target. Ther..

[27] Ciuro M., Sangiorgio M., Cacciato V., Cantone G., Fichera C., Salvatorelli L., Magro G., Leanza G., Vecchio M., Valle M.S. (2024). Mitigating the Functional Deficit after Neurotoxic Motoneuronal Loss by an Inhibitor of Mitochondrial Fission. Int. J. Mol. Sci..

[28] Bordt E.A., Clerc P., Roelofs B.A., Saladino A.J., Tretter L., Adam-Vizi V., Cherok E., Khalil A., Yadava N., Ge S.X. (2017). The Putative Drp1 Inhibitor Mdivi-1 is a Reversible Mitochondrial Complex I Inhibitor that Modulates Reactive Oxygen Species. Dev. Cell.

[29] Marx N., Ritter N., Disse P., Seebohm G., Busch K.B. (2024). Detailed Analysis of Mdivi-1 Effects on Complex I and Respiratory Supercomplex Assembly. Sci. Rep..

[30] Luo G., Yi J., Ma C., Xiao Y., Yi F., Yu T., Zhou J. (2013). Defective Mitochondrial Dynamics is an Early Event in Skeletal Muscle of an Amyotrophic Lateral Sclerosis Mouse Model. PLoS ONE.

[31] Wang H., Yi J., Li X., Xiao Y., Dhakal K., Zhou J. (2018). ALS-Associated Mutation SOD1G93A Leads to Abnormal Mitochondrial Dynamics in Osteocytes. Bone.

[32] Choi S.Y., Lee J.H., Chung A.Y., Jo Y., Shin J.H., Park H.C., Kim H., Lopez-Gonzalez R., Ryu J.R., Sun W. (2020). Prevention of Mitochondrial Impairment by Inhibition of Protein Phosphatase 1 Activity in Amyotrophic Lateral Sclerosis. Cell Death Dis..

[33] Gurney M.E., Pu H., Chiu A.Y., Dal Canto M.C., Polchow C.Y., Alexander D.D., Caliendo J., Hentati A., Kwon Y.W., Deng H.X. (1994). Motor Neuron Degeneration in Mice that Express a Human Cu,Zn Superoxide Dismutase Mutation. Science.

[34] Turner B.J., Talbot K. (2008). Transgenics, Toxicity and Therapeutics in Rodent Models of Mutant SOD1-Mediated Familial ALS. Prog. Neurobiol..

[35] Crowe A.R., Yue W. (2019). Semi-quantitative Determination of Protein Expression using Immunohistochemistry Staining and Analysis: An Integrated Protocol. Bio Protoc..

[36] Cassidy-Stone A., Chipuk J.E., Ingerman E., Song C., Yoo C., Kuwana T., Kurth M.J., Shaw J.T., Hinshaw J.E., Green D.R. (2008). Chemical Inhibition of the Mitochondrial Division Dynamin Reveals its Role in Bax/Bak-Dependent Mitochondrial Outer Membrane Permeabilization. Dev. Cell.

[37] Zhou K., Yang H.Y., Tang P.Y., Liu W., Luo Y.J., Lv B., Yin J., Jiang T., Chen J., Cai W.H. (2018). Mitochondrial Division Inhibitor 1 Protects Cortical Neurons from Excitotoxicity: A Mechanistic Pathway. Neural Regen. Res..

[38] Kim H., Lee J.Y., Park K.J., Kim W.H., Roh G.S. (2016). A Mitochondrial Division Inhibitor, Mdivi-1, Inhibits Mitochondrial Fragmentation and Attenuates Kainic Acid-Induced Hippocampal Cell Death. BMC Neurosci..

[39] Wang J., Wang P., Li S., Wang S., Li Y., Liang N., Wang M. (2014). Mdivi-1 Prevents Apoptosis Induced by Ischemia-Reperfusion Injury in Primary Hippocampal Cells via Inhibition of Reactive Oxygen Species-Activated Mitochondrial Pathway. J. Stroke Cerebrovasc. Dis..

[40] Zhang N., Wang S., Li Y., Che L., Zhao Q. (2013). A Selective Inhibitor of Drp1, Mdivi-1, Acts Against Cerebral Ischemia/Reperfusion Injury via an Anti-Apoptotic Pathway in Rats. Neurosci. Lett..

[41] Ferraiuolo L., Kirby J., Grierson A.J., Sendtner M., Shaw P.J. (2011). Molecular Pathways of Motor Neuron Injury in Amyotrophic Lateral Sclerosis. Nat. Rev. Neurol..

[42] Goutman S.A., Hardiman O., Al-Chalabi A., Chiò A., Savelieff M.G., Kiernan M.C., Feldman E.L. (2022). Emerging Insights Into the Complex Genetics and Pathophysiology of Amyotrophic Lateral Sclerosis. Lancet Neurol..

[43] Gulino R., Perciavalle V., Gulisano M. (2010). Expression of Cell Fate Determinants and Plastic Changes after Neurotoxic Lesion of Adult Mice Spinal Cord by Cholera Toxin-B Saporin. Eur. J. Neurosci..

[44] Gulino R., Parenti R., Gulisano M. (2015). Novel Mechanisms of Spinal Cord Plasticity in a Mouse Model of Motoneuron Disease. BioMed Res. Int..

[45] Gulino R., Gulisano M. (2013). Noggin and Sonic Hedgehog are Involved in Compensatory Changes within the Motoneuron-Depleted Mouse Spinal Cord. J. Neurol. Sci..

[46] Gulino R., Vicario N., Giunta M.A.S., Spoto G., Calabrese G., Vecchio M., Gulisano M., Leanza G., Parenti R. (2019). Neuromuscular Plasticity in a Mouse Neurotoxic Model of Spinal Motoneuronal Loss. Int. J. Mol. Sci..

[47] Gómez-Pinilla F., So V., Kesslak J.P. (2001). Spatial Learning Induces Neurotrophin Receptor and Synapsin I in the Hippocampus. Brain Res..

[48] Gulino R., Dimartino M., Casabona A., Lombardo S.A., Perciavalle V. (2007). Synaptic Plasticity Modulates the Spontaneous Recovery of Locomotion After Spinal Cord Hemisection. Neurosci. Res..

[49] Dukkipati S.S., Garrett T.L., Elbasiouny S.M. (2018). The Vulnerability of Spinal Motoneurons and Soma Size Plasticity in a Mouse Model of Amyotrophic Lateral Sclerosis. J. Physiol..

[50] Giusto E., Codrich M., de Leo G., Francardo V., Coradazzi M., Parenti R., Gulisano M., Vicario N., Gulino R., Leanza G. (2020). Compensatory Changes in Degenerating Spinal Motoneurons Sustain Functional Sparing in the SOD1-G93A Mouse Model of Amyotrophic Lateral Sclerosis. J. Comp. Neurol..

[51] Gulino R. (2023). Synaptic Dysfunction and Plasticity in Amyotrophic Lateral Sclerosis. Int. J. Mol. Sci..

[52] Taylor H.B.C., Jeans A.F. (2021). Friend or Foe? The Varied Faces of Homeostatic Synaptic Plasticity in Neurodegenerative Disease. Front. Cell. Neurosci..

[53] Ciuro M., Sangiorgio M., Leanza G., Gulino R. (2026). Neuroinflammation and Oxidative Stress in SOD1 Animal Models of ALS: A Meta-analysis Study of Their Effects on Disease Onset and Progression. Mol. Neurobiol..

[54] Ciuro M., Sangiorgio M., Leanza G., Gulino R. (2022). A Meta-Analysis Study of SOD1-Mutant Mouse Models of ALS to Analyse the Determinants of Disease Onset and Progression. Int. J. Mol. Sci..

[55] Cho H.M., Ryu J.R., Jo Y., Seo T.W., Choi Y.N., Kim J.H., Chung J.M., Cho B., Kang H.C., Yu S.W. (2019). Drp1-Zip1 Interaction Regulates Mitochondrial Quality Surveillance System. Mol. Cell.

[56] Gomes L.C., Di Benedetto G., Scorrano L. (2011). During Autophagy Mitochondria Elongate, are Spared from Degradation and Sustain Cell Viability. Nat. Cell Biol..

[57] Gómez-Oliver F., Fernández de la Rosa R., Brackhan M., Bascuñana P., Pozo M.Á., García-García L. (2025). Inhibition of Astrocyte Reactivity by Mdivi-1 After Status Epilepticus in Rats Exacerbates Microglia-Mediated Neuroinflammation and Impairs Limbic-Cortical Glucose Metabolism. Biomolecules.

[58] Moloney E.B., de Winter F., Verhaagen J. (2014). ALS as a Distal Axonopathy: Molecular Mechanisms Affecting Neuromuscular Junction Stability in the Presymptomatic Stages of the Disease. Front. Neurosci..

[59] Martineau É., Di Polo A., Vande Velde C., Robitaille R. (2018). Dynamic Neuromuscular Remodeling Precedes Motor-Unit Loss in a Mouse Model of ALS. eLife.

[60] Fogarty M.J. (2018). Driven to Decay: Excitability and Synaptic Abnormalities in Amyotrophic Lateral Sclerosis. Brain Res. Bull..

[61] Walker A.K., Soo K.Y., Sundaramoorthy V., Parakh S., Ma Y., Farg M.A., Wallace R.H., Crouch P.J., Turner B.J., Horne M.K. (2013). ALS-Associated TDP-43 Induces Endoplasmic Reticulum Stress, which Drives Cytoplasmic TDP-43 Accumulation and Stress Granule Formation. PLoS ONE.

[62] Neumann M., Sampathu D.M., Kwong L.K., Truax A.C., Micsenyi M.C., Chou T.T., Bruce J., Schuck T., Grossman M., Clark C.M. (2006). Ubiquitinated TDP-43 in Frontotemporal Lobar Degeneration and Amyotrophic Lateral Sclerosis. Science.

[63] Prasad A., Bharathi V., Sivalingam V., Girdhar A., Patel B.K. (2019). Molecular Mechanisms of TDP-43 Misfolding and Pathology in Amyotrophic Lateral Sclerosis. Front. Mol. Neurosci..

[64] Grohm J., Kim S.W., Mamrak U., Tobaben S., Cassidy-Stone A., Nunnari J., Plesnila N., Culmsee C. (2012). Inhibition of Drp1 Provides Neuroprotection in Vitro and in Vivo. Cell Death Differ..

[65] Baek S.H., Park S.J., Jeong J.I., Kim S.H., Han J., Kyung J.W., Baik S.H., Choi Y., Choi B.Y., Park J.S. (2017). Inhibition of Drp1 Ameliorates Synaptic Depression, Aβ Deposition, and Cognitive Impairment in an Alzheimer’s Disease Model. J. Neurosci..

[66] Cho B., Choi S.Y., Cho H.M., Kim H.J., Sun W. (2013). Physiological and Pathological Significance of Dynamin-Related Protein 1 (Drp1)-Dependent Mitochondrial Fission in the Nervous System. Exp. Neurobiol..

[67] Duranti E. (2025). The Role of Skeletal Muscle in Amyotrophic Lateral Sclerosis: State of the Art 2025. Muscles.

[68] Chargé S.B., Rudnicki M.A. (2004). Cellular and Molecular Regulation of Muscle Regeneration. Physiol. Rev..

[69] Dumont N.A., Wang Y.X., von Maltzahn J., Pasut A., Bentzinger C.F., Brun C.E., Rudnicki M.A. (2015). Dystrophin Expression in Muscle Stem Cells Regulates their Polarity and Asymmetric Division. Nat. Med..

[70] Günther R., Pal A., Williams C., Zimyanin V.L., Liehr M., von Neubeck C., Krause M., Parab M.G., Petri S., Kalmbach N. (2022). Alteration of Mitochondrial Integrity as Upstream Event in the Pathophysiology of SOD1-ALS. Cells.

